# Impact of Obesity on Influenza A Virus Pathogenesis, Immune Response, and Evolution

**DOI:** 10.3389/fimmu.2019.01071

**Published:** 2019-05-10

**Authors:** Rebekah Honce, Stacey Schultz-Cherry

**Affiliations:** ^1^Department of Infectious Diseases, St. Jude Children's Research Hospital, Memphis, TN, United States; ^2^Integrated Program in Biomedical Sciences, Department of Microbiology, Immunology, and Biochemistry, University of Tennessee Health Science Center, Memphis, TN, United States

**Keywords:** influenza, obesity, pathogenesis, evolution, immunity

## Abstract

With the rising prevalence of obesity has come an increasing awareness of its impact on communicable disease. As a consequence of the 2009 H1N1 influenza A virus pandemic, obesity was identified for the first time as a risk factor for increased disease severity and mortality in infected individuals. Over-nutrition that results in obesity causes a chronic state of meta-inflammation with systemic implications for immunity. Obese hosts exhibit delayed and blunted antiviral responses to influenza virus infection, and they experience poor recovery from the disease. Furthermore, the efficacy of antivirals and vaccines is reduced in this population and obesity may also play a role in altering the viral life cycle, thus complementing the already weakened immune response and leading to severe pathogenesis. Case studies and basic research in human cohorts and animal models have highlighted the prolonged viral shed in the obese host, as well as a microenvironment that permits the emergence of virulent minor variants. This review focuses on influenza A virus pathogenesis in the obese host, and on the impact of obesity on the antiviral response, viral shed, and viral evolution. We comprehensively analyze the recent literature on how and why viral pathogenesis is altered in the obese host along with the impact of the altered host and pathogenic state on viral evolutionary dynamics in multiple models. Finally, we summarized the effectiveness of current vaccines and antivirals in this populations and the questions that remain to be answered. If current trends continue, nearly 50% of the worldwide population is projected to be obese by 2050. This population will have a growing impact on both non-communicable and communicable diseases and may affect global evolutionary trends of influenza virus.

## Introduction

Obesity rates have nearly tripled worldwide since 1975. Approximately 1.9 billion people are overweight and over 650 million are obese, defined as having a body mass index (BMI) of 25 to 30 and >30, respectively, which translates to nearly 45% of adults worldwide (1, 2). The obesity-induced inflammatory state has systemic implications for individual and global public health. It is a well-identified risk factor for increased mortality due to heightened rates of heart disease, certain cancers, and musculoskeletal disorders (3). Overnutrition, as well as undernutrition, has been cited as an important factor in the body's response to infection for centuries (4–6). More recently, the impact of obesity on communicable diseases has been appreciated. During the 2009 influenza A virus (IAV) H1N1 pandemic, a plethora of epidemiologic studies revealed obesity to be an independent risk factor for severe disease (7, 8). In initial retrospective studies of laboratory-confirmed H1N1 cases after the 2009 pandemic, obesity was identified as a risk factor for hospitalization, the need for mechanical ventilation, and mortality upon infection (9–11).

Influenza is a potentially severe respiratory infection caused by the influenza virus. Most human cases are caused by H1N1 and H3N2 IAV strains (12, 13). Several case studies of severe and fatal IAV infections have identified possible effects of obesity on disease progression; these effects include extensive viral replication in the deep lung, progression to viral pneumonia, and prolonged and increased viral shedding (14–16). However, these studies neglected to determine the causality between obesity and severe IAV pathogenesis. Studies in mouse models of obesity, including leptin-deficient (OB) and leptin-receptor-deficient (DB) genetically obese models as well as the high-fat diet-induced-obese (DIO) model (Table 1) have identified several immunological mechanisms for the increased pathogenesis and mortality that mirrors what has been seen in humans (26–29). Advances in obese ferret models and human primary cell culture are enabling better translational studies of the mechanisms behind the increased disease severity, viral life cycle alterations, and evolutionary dynamics due to the obesogenic environment (30). In this review, we analyze the current literature for information on the impact of obesity on influenza virus pathogenesis, the immune responses to the virus and the transmission dynamics of IAV including viral shedding and evolution.

**Table 1:** Description of commonly used mouse models of obesity.

**Model**	**ID**	**Genetics**	**Weight^a^**	**Diet and behavior**	**References**
Genetic leptin knockout	OB	Most commonly on C57BL/6 background; spontaneous recessive, homozygous *Lep^*ob*^* nonsense mutation	45 g	Normal chow; hyperphagic due to loss of appetite control and satiety	(17, 18)
Genetic leptin receptor knockout	DB	Commonly on C57BL/Ks or C57BL/6J backgrounds; spontaneous mutant in *Lepr^*db*^* allele causing abnormal splicing	40 g	Normal chow; hyperphagic due to loss of leptin receptor signal transduction	(19, 20)
Diet-induced	DIO	Any background, commonly C57BL/6J; some strains more susceptible than others	35 g	High-fat diet; exhibits typical eating patterns	(21–25)
Control	LN/WT	Any matched genetic background	25 g	Either low-fat diet (LN) or regular chow; diet choice may alter results	(21, 25)

a *Typical weight in grams for an 8-week-old male*.

## Pathogenesis and Resolution of IAV Infection

IAV infection is characterized by fever, myalgia, rhinorrhea, sore throat and sneezing. Symptoms peak 3–5 days post infection (p.i.), with viral shedding peaking at day 2–3 p.i. (31). Most human IAV infections are limited to the upper respiratory tract, including the nasal, tracheal and often bronchial epithelium. In more severe cases, there is infection of the lower respiratory tract (LRT) including the lung occurs, often with severe sequelae requiring hospitalization (32). Development of viral pneumonia and secondary bacterial infections can lead to acute lung injury (ALI), acute respiratory distress syndrome (ARDS), and eventual death (32). Progression of the infection to the LRT and severe sequelae are more common in the obese population, leading to poorer infection resolution and recovery than is seen in non-obese patients.

### Lung Pathology and Infection Outcomes

After the 2009 H1N1 pandemic, retrospective studies across the globe found obesity to be comorbid with influenza in nearly one-third of hospitalized patients as well as in fatal cases (33–35). In both pandemic and non-pandemic influenza seasons, obesity increased the risk of hospitalization for laboratory-confirmed IAV infection, with increasing BMI increasing the odds ratios (36, 37). The susceptibility extended to heightened disease severity, with obese children and adults experiencing increased morbidity and mortality during LRT infections, including comorbid secondary infections and a risk of ARDS (38, 39). Obesity was shown to increase both the length of stay in intensive care and the need for mechanical ventilation (40). Most strikingly, severe obesity resulted in a two-times greater risk of death upon IAV infection and hospitalization due to the infection, with moderate obesity also increasing the risks (41, 42). In other high-risk populations such as pregnant and post-partum women, obesity further increased the risk of IAV infection (43).

Upon infection with IAV, the viral replication process as well as the pro-inflammatory, antiviral immune response, damages the respiratory epithelium and recovery requires proper clearance and remodeling of the damaged surfaces. Increased lung damage, pulmonary edema, cellularity, inflammatory response, and immunopathology is evident in DIO and OB mice as compared to wild-type (WT) mice inoculated with IAV in both naïve and vaccine challenge experiments (26, 27, 44–48). The few comparative studies for which no difference in lung pathology was reported used relatively high lethal doses, highlighting the importance of taking into account the viral stock preparation and the means of administration when comparing host responses and pathogenesis (49).

In severe cases, IAV infection can cause a break-down of the respiratory epithelium, leading to fluid influx to the airway space (32). Obese mice are more likely than are lean (LN) mice to have increased lung permeability during infection. Using Evan's blue dye, Karlsson et al. found that at day 7 p.i., OB mice showed a significantly greater increase in permeability when compared to LN mice (48). This finding was confirmed by the increased albumin levels in bronchoalveolar lavage fluid (BALF) at days 5–8 p.i., in both OB and DIO mice, showing that there is increased protein leakage from the lung into the BALF (27, 48, 50). The increased lung permeability is coupled with an increase in lung edema and oxidative stress upon IAV infection, emphasizing the multiple etiologies of increased lung pathology in the obese host (27, 46, 49). Infection resolution requires the repair of the damaged epithelial surface, but OB and DIO hosts are impaired in wound repair (27). Reduced cellular proliferation as determined through decreased KI-67 staining is evident in OB and DIO mouse lung sections at days 6 and 14 p.i, leaving the lung susceptible to secondary infections and eventual ARDS (27).

The heightened immunopathology and poor wound recovery in OB and DIO mice result in increased mortality. IAV strains H3N2 and H1N1 induce greater mortality in OB and DIO mice than in WT C57BL/6 mice, regardless of their respective vaccine histories (26, 27, 47, 50). This is also true for a viral-bacterial co-infection model. DIO and OB mice inoculated with PR8 IAV, CA/09 IAV, seasonal H3N2 virus, or influenza B virus and challenged with *Streptococcus pneumoniae* at day 7 post influenza infection had increased mortality when compared to controls (48).

### Viral Load and Spread in Respiratory Epithelia

The increased incidence of ALI and ARDS in hospitalized obese patients may be due to increased viral spread to the LRT and alveolar region, thus resulting in impaired lung function and gas exchange (38). Limited case studies that list obesity as a comorbidity reference heightened viral replication and extensive hemorrhage in the alveoli leading to increased disease severity (16, 51). Continued investigation using *ex vivo* human systems as well as following naturally occurring infections in cohorts of obese and lean patients can help determine how the data gleaned from mouse models translates to human infection, as well as how other comorbidities such as metabolic syndrome, chronic disease, age, and gender will affect the pathogenesis of IAV (3, 52, 53).

Although some studies have demonstrated higher viral titers in obese mice than in non-obese animals, others have found no such difference (27, 44). In a viral-bacterial co-infection model, there was no difference in the influenza viral load between obese and WT mice at peak disease, but obese mice had higher viral titers at later timepoints when compared to WT controls (48). Similarly, the viral titers in OB and DIO mice infected with H1N1 viruses were no different to the titers in WT mice at peak infection at days 3 and 6 p.i., but the obese mice had prolonged infections (27, 54). Titration of the virus in lung homogenates showed that WT animals had undetectable levels of virus by day 10 p.i. whereas OB mice showed no discernable decrease in viral titer (54). Conversely, some reports have suggested that DIO mice have higher viral titers early in infection with no change at later timepoints post-infection (44, 49). The disparities between these reports may be due to differences in the inoculation method, dose, heterogeneity of influenza viral strains, or viral stock preparations. Nevertheless, OB mice experience worse outcomes after infection independent of increased viral titers.

Obese mice exhibit increased viral spread to the LRT. More viral antigen was present in the bronchiolar and alveolar regions in DIO mice inoculated with H1N1 virus than in the corresponding regions of infected control animals (55). In the viral-bacterial co-infection model, OB mice inoculated with a fluorescent reporter virus showed increased viral spread in the nasopharynx, trachea, and lung at day 8 and 9 p.i., as determined through live-animal imaging, along with more extensive areas of active viral infection at days 7 and 9 p.i., as determined by nucleoprotein staining of sectioned lung tissue (48). Excised lungs from OB mice showed this increased viral spread to be present as early as day 3 p.i (54). The culmination of severe lung pathology and increased viral spread leads to increased mortality in obese mice due to influenza infection and severe sequelae (27).

Systemic immune deficits and obesity-related poor pulmonary mechanics contribute to the observed increased susceptibility of obese hosts to IAV (56); however, *ex vivo* studies with primary human respiratory epithelial cells have revealed intrinsic cellular differences in viral replication in obese and lean subjects (57, 58). In limited studies with alveolar epithelial cells (AECs), Huang et al. showed that H7N9 infection of obese-derived cells resulted in a greater increase in viral RNA production from 24 to 72 h post infection (h.p.i.) than was seen in infected of lean-derived cells (58). These results support the limited data demonstrating that obese AECs are more susceptible to infection (57). Further studies are warranted to elucidate the mechanism behind this increased susceptibility at the epithelial cell level.

## Host Antiviral Response to Influenza Infection

**Figure 1:**
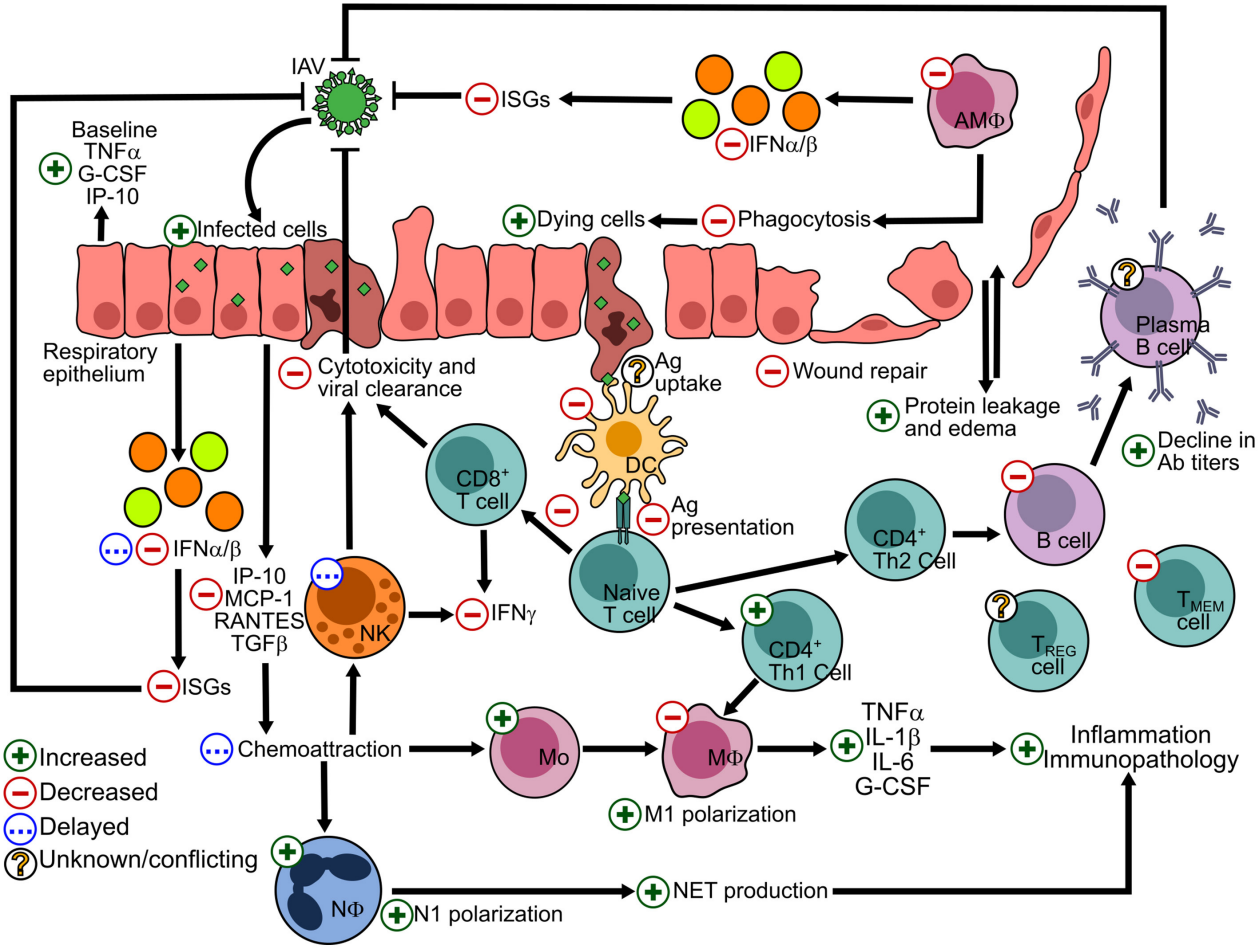
Alterations to the host response to IAV in the lung epithelium due to the obese state. The effects of obesity on antiviral processes are summarized by a green + symbol indicating and increased number or process; a red—sign indicating a decreased number or process; a blue ellipses (…) indicating a delayed response; and a yellow interrogation mark (?) indicating conflicting or scarce literature. IFN, interferon; ISGs, interferon-stimulated genes; Ab, antibody; Ag, antigen; Adapted and updated from references (28, 60).

Obesity results in a dampened immune response to infectious agents, leading to poorer outcomes post-infection (3, 28, 59). Systemic alterations to antiviral immunity, including both the innate and adaptive responses, have been described for IAV infection of the respiratory epithelium (Figure 1). Baseline alterations in the obese lung environment affect the viral pathogenesis and immune response and leave the lung susceptible to increased viral spread and secondary infections due to the poor induction of antiviral immunity.

### Innate Immunity

Non-specific and quick-acting innate immune defenses provide the initial host response to IAV in the lung epithelium (61). An altered chemokine and cytokine milieu is characteristic of obese hosts and contributes to the meta-inflammation seen in this population (62, 63). Increased visceral and omental adiposity results in an efflux of pro-inflammatory cytokines that influence systemic cellular processes (56, 64–66). The baseline and post-IAV infection chemokine and cytokine alterations in mouse models of obesity are outlined in Table 2.

**Table 2:** Obesity-related alterations in chemokine, cytokine, and interferon stimulated genes at baseline and during influenza infection in murine models of obesity.

**Analyte**	**Model**	**Effect at baseline**	**Effect during infection**	**References**
TGFβ	DIO	↓ Lung concentration; ↑ lung mRNA expression; ↑ BALF concentration	↓ Lung concentration	(27, 67)
TNFα	DIO	↑ Plasma concentration; ↑ lung mRNA expression; ↑ BALF concentration	↑ BALF concentration at 8 dpi; delayed lung mRNA expression ↑ at 3 and 6 dpi; delayed BALF concentration 3 and 8 dpi; ↓ lung concentration fold increase at 1 and 3 dpi; ↑ circulating concentration fold increase at 3 dpi	(26, 46, 55, 67–70)
	DB		↑ BALF concentration at 4 dpi	
G-CSF	DIO	↑ Lung concentration	↓ Lung concentration at 3 dpi; delayed BALF concentration ↑ at 3 and 8 dpi	(27, 70)
IL-1β	DIO		↓ Lung mRNA expression at 3 dpi; ↓ BALF concentration at 3 dpi; ↓ lung concentration fold increase at 1 and 3 dpi; ↑ lung concentration at 4 dpi; ↑ circulating concentration from baseline at 3 dpi	(55, 70–72)
IL-2	DIO		↓ LN mRNA expression at 3 and 7 dpi	(26)
IL-5	DIO		↓ BALF concentration at 3 dpi	(70)
IL-6	DIO		Fewer macrophages producing ↑ at 3 dpi; delayed lung mRNA expression at 3 and 6 dpi, delayed BALF concentration ↑ at 3 and 8 dpi, ↑ lung and serum concentration at 4 dpi	(26, 70–72)
IL-12	DIO		↓ LN mRNA expression at 3 and 7 dpi	(71)
IL-12p70	DIO		↓ BALF concentration at 3 dpi	(70)
IL-13	DIO		Delayed BALF concentration at 3 and 8 dpi	(70)
Leptin	DIO	↑ Serum concentration; ↑ lung mRNA expression	↑ Serum concentration ↑ at 2, 4, and 6 dpi; ↓ circulating concentration fold increase at 3 dpi	(26, 44, 55, 67, 72)
Adiponectin	DIO	↓ Serum concentration; ↓ lung mRNA expression	↓ Serum concentration at 2 and 6 dpi	(26, 67, 72)
CXCL1/KC	DIO	↑ Plasma concentration	↑ BALF concentration at 8 dpi	(46, 50, 68)
	OB		↑ BALF concentration increase at 8 dpi	
CXCL2/ MIP2α	DIO	↑ Plasma concentration	↑ Lung concentration at 4 dpi	(68, 72)
CXCL10/ IP-10	DIO	↑ Plasma concentration	↓ Lung mRNA expression at 1 dpi; delayed BALF ↑ concentration at 3 and 8 dpi	(68, 70)
CCL2/ MCP-1	DIO		↑ BALF concentration at 8 dpi; ↓ lung mRNA expression at 3 dpi	(46, 71)
CCL3/ MIP1α	DIO		↓ BALF concentration at 3 dpi; ↑ lung concentration at 4 dpi	(70, 72)
CCL5/ RANTES	DIO	↑ Plasma concentration	Delayed lung mRNA expression at 3 and 6 dpi; ↓ BALF concentration at 3 and 8 dpi	(68, 70, 72)
CCL7/ MCP3	DIO	↑ Plasma concentration		(68)
CCL11/ Eotaxin	DIO		Delayed BALF concentration 3 and at 8 dpi	(70)
IFNα	DIO		↓ Lung mRNA expression at 2 dpi	(26)
IFNβ	DIO		↓ Lung mRNA expression at 2 and 3 dpi	(26)
IFNγ	DIO		↑ Circulating concentration fold increase at 3 dpi	
Irf7	OB	↓ Alveolar macrophage mRNA expression	↓ Alveolar macrophage mRNA expression at 1 dpi; ↓ lung mRNA expression fold-increase at 1 dpi	(54, 70)
Oas1a	DIO		↓ Lung mRNA expression fold-increase at 1 dpi	(70)
Oas1g	OB	↓ Alveolar macrophage mRNA expression; ↓ epithelial cell mRNA expression	↓ Alveolar macrophage mRNA expression at 1 dpi	(54)
Ifit1	OB	↓ Alveolar macrophage mRNA expression	↓ Alveolar macrophage mRNA expression at 1 dpi	(54)
Mx2	DIO		↓ Lung mRNA expression fold-increase at 1 dpi	(70)

*↑increased, ↓decreased, LN, lymph node; BALF, bronchial alveolar lavage fluid. Fold increase is measured as the fold increase from baseline of uninfected obese mice; delayed increase in expression or concentration relates to a delayed increase from baseline as compared to the immune response in matched WT mice*.

In DIO and OB mice, baseline alterations in systemic and localized chemokines and cytokines results from increased visceral and omental adiposity (66, 73). The adipose tissue of OB and DIO mice produces heightened levels of several adipokines including MCP-1, TNF-α, and IL-6, as well as the cytokine transforming growth factor-β (TGF-β), all of which have systemic impacts (64, 66, 74). Two key adipokines that are intimately tied to obese status are leptin and adiponectin (75–77). Higher serum leptin and lower adiponectin levels due to obesity may contribute to the heightened pro-inflammatory cytokine production, as well as to the poor responsiveness to infection stimuli in DIO mice (78). Early in IAV infection of DIO mice, the expression of IL-6, TNFα, and type I interferons (IFNs) is delayed and reduced compared to that in LN mice, and differential regulation of IL-2 and IL-12 is detected in lung homogenates, whereas later in infection there is increased inflammation in DIO mice as compared to LN mice later in infection (26, 55, 64). This is converse to the baseline state in the lungs of obese mice, which is characterized by high expression of pro-inflammatory cytokines and chemokines (44). Differential regulation of the antiviral cytokines and IFN response upon IAV infection in obese mice may contribute to the poor adaptive responses as discussed below (26, 55, 71).

Obese adult humans, like mouse models, have elevated circulating pro-inflammatory cytokines as well as increased leptin and reduced adiponectin levels, leading to a systemic leptin-resistance and effects on the baseline immune status (79, 80). Fewer studies have investigated the innate antiviral responses. In studies with primary human epithelial cells, cells from obese individuals displayed a smaller change in the levels of IL-8, IL-1β, and IP-10 levels at 72 h.p.i., which is consistent with the delayed and blunted cytokine response seen in obese rodent models (58). Future studies should continue to explore the epithelial-specific responses to influenza infection in primary cells of lean and obese individuals, as the respiratory epithelium is the primary site of influenza infection and replication.

Systemic alterations in cytokine levels upon infection may be due to upregulated expression of suppressor of cytokine signaling (SOCS) proteins. SOCS proteins are involved in the negative regulation of JAK-STAT signaling and in the induction of type I and type III IFNs and pro-inflammatory cytokines (81–83). In DIO mice, there is upregulated expression of SOCS1 and SOCS3 mRNA expression in the lungs (78). Similar upregulation of SOCS expression has been seen in PBMCs isolated from lean and obese humans. Reduced type I IFN production from toll-like receptor (TLR)-stimulated PBMCs was concordant with SOCS3 overexpression in cells derived from obese subjects (84). Overexpression of SOCS3 and SOCS1 proteins was evident at baseline in PBMCs from obese subjects, but stimulation with TLR3 and TLR7 specific agonists caused no concordant increase in expression as is typical in cells derived from lean individuals (85). These alterations may be tied to leptin resistance, as leptin uses downstream signal transduction pathways similar to those for antiviral chemokines and cytokines (86, 87). Changes in lipids and lipid-derived mediators also affect the response to IAV in obese hosts. The impact of obesity on the lung metabolome contributes to the increased severity of IAV disease in DIO mice, which may be ameliorated through exogenous administration of statins and acetaminophen (88, 89).

### Antigen Presentation and Phagocytosis

The bridge between non-specific innate immunity and antigen-specific adaptive immunity relies on appropriate actions of immune cells from both the lymphoid and myeloid lineages (90). There are more leukocytes in the lungs of DIO mice than in those of WT mice (91). Various effects of obesity on these leukocyte subpopulations including macrophages, dendritic cells, natural killer (NK) cells, and neutrophils have been described and the reports are often contradictory (28, 61, 92, 93). Here, we will focus on influenza-specific investigations and on the functional changes in these immune cells in the respiratory system (Table 3).

**Table 3:** Immune cell subsets in the obese lung environment at baseline and during influenza infection in murine models of obesity.

**Cell type**		**Model**	**Impact at baseline**	**Impact during infection**	**References**
Monocytes		DIO		↑ BALF infiltration at 3, 4, 6, and 14 dpi	(27)
		OB		↑ BALF infiltration at 3, 6,	
				and 14 dpi	
		DB		↑ BALF infiltration at 4 dpi	
Macrophages		DIO	↑ M1 polarization; ↓ CD86+ and activation; ↓ migration to lung	↓ BALF at 4 dpi	(46)
Alveolar Macrophages		OB	↓ Type I IFN receptor signaling	↓ IFN mRNA expression, ↓ IFNAR signaling; Δ Numbers in BALF at 4 dpi; ↓ Numbers in BALF at 7 dpi	(46, 48)
			↑ BALF number		
Interstitial Macrophages		DIO		Δ Numbers in BALF at 4 dpi	
Natural Killer cells		DIO	Δ Lung number	↑ BALF infiltration at 6 and 14 dpi; ↓ cytotoxicity; ↓ Lung number at 3 dpi	(26, 27, 46)
		OB	↑ Lung number	↑ BALF infiltration at 6 and 14 dpi; ↓ BALF infiltration at 4 dpi	
		DB		↓ BALF infiltration at 4 dpi	
Dendritic cells		DIO	↓ Lung pDC number	↓ Antigen presentation, ↓ Induction of T cell proliferation; ↓ lung DN DC and pDC number at 3 dpi; ↑ LN migration	(71)
Neutrophils		DIO	↑ N1 polarization	↑ NET production, ↑ BALF infiltration at 6 and 14 dpi	(27, 48, 49)
		OB		↑ BALF infiltration at 3, 6, and 14 dpi; ↓ BALF infiltration at 7 dpi	
		DB		↑ BALF infiltration at 4 dpi	
B cells		DIO		↑ Circulating IgG; ↓ mature bone marrow B cells; ↓ cross-reactive H1N1 and PR8 antibodies at 5, 8, and 14 dpi	(50, 94)
T cells	CD8+	DIO	↑ OCR:ECAR ratios	↓ Lung at 7 dpi; ↑ cross-reactive CD8+ cells at 5 dpi	(46, 50, 68, 71, 78, 95)
	CD4+		↑ OCR:ECAR ratios	↑ Lung at 5 dpi in heterologous challenge; ↓ lung at 3, 4 dpi, and 84 dpi; ↓ BALF at 8 dpi	
	T_REG_		↓ Lung numbers		
	T_MEM_			Δ Primary T_MEM_ at 30 dpi; ↑ and T_CM_ at 5 dpi in heterologous challenge; ↓ lung T_MEM_ at 3 and 7 dpi; ↓ T_REG_ suppression of lean T_EM_	

*↑increased, ↓decreased, Δ no change/equal; BALF, bronchiolar lavage fluid; DN DC, double negative dendritic cell; pDC, plasmacytoid dendritic cell; T_REG_, regulatory T cell; T_MEM_, memory T cells; T_CM_, central memory T cell; T_EM_, effector memory T cell*.

The systemic pro-inflammatory state of the obese host extends to the lung microenvironment. Macrophages collected from bone marrow and blood of OB and DIO mice show a polarization toward an M1 pro-inflammatory phenotype (91, 96). This is compounded by the impaired migration of macrophages to lung alveoli during infection, which could lead to poor clearance of the invading pathogen (46, 91). In DIO and OB mice, lung resident alveolar macrophages are reduced in number as a result of infection, and those that remain show a reduction in the expression of type I IFN receptor and in IFN-stimulated gene expression when compared to WT mice (26, 54). These changes may be due in part to the obesity-related chronic inflammation, as a chronically inflamed mouse model shows similarly reduced macrophage activation and blunted pro-inflammatory cytokine production upon macrophage stimulation (97).

The poor vaccine response that is characteristic of the obese host (and is further discussed below) may be partially due to suboptimal macrophage functionality and maturation. Although phagocytosis is increased in peritoneal macrophages isolated from DIO mice, macrophages derived from obese individuals show reduced activation as measured by CD86 expression before and after stimulation *ex vivo*, impairing their ability to activate effector cells of the immune system (98). This finding is further supported by the increased presence of immature monocytes but equal presence of effector alveolar and interstitial macrophages in BALF collected from DB mice at day 4 p.i (69). Continued studies of the physiologic function of lung-resident and migratory macrophages will increase our understanding of the mechanism behind poor induction of the immune and vaccine response.

Dendritic cells (DCs) prime the T cell response of the immune system through presentation of phagocytosed antigens. Several studies have shown that the phagocytotic function and activation of DCs and other antigen presenting cells remain sufficient in the obese host (29, 99), whereas another study suggests that the overall number of DCs and antigen presentation is impaired in DCs from lungs of DIO mice (71). The authors of the latter study argue that the reduction in antigen presentation by DCs fails to instruct the T cell response and is related to the elevated IL-6-driven pro-inflammatory state of the DIO mouse lung (71). Further studies have shown that in OB and DIO mice, poorly activated DCs fail to induce T cell proliferation, possibly because similar pro-inflammatory signals blunt their maturation (66, 100, 101). In human cohorts, obese patients have reduced number of circulating DCs and those remaining are less responsive to *ex vivo* stimulation with TLR agonists than are those of non-obese patients; however, further studies are needed to discern whether this observation extends to IAV infection (102).

Neutrophils also show a pro-inflammatory N1 phenotype in DIO mice, and those from obese humans show increased production of inflammatory free radicals upon stimulation *ex vivo* (80, 91). Lethally challenging DIO mice with PR8 virus results in the production of more neutrophil extracellular traps than are seen in LN mice, and an increase in neutrophil migration is seen at day 3 p.i. in the lungs of OB mice, at days 6 and 14 p.i. in OB and DIO mice, and at day 4 p.i. in the DB BALF (27, 49, 69). This may compound the severity of the observed lung pathology as a result of the increased release of cytotoxic proteins and the development of ALI (27, 46, 103). Conversely, NK cells are decreased in DIO mice as well as in overweight and obese humans but increased in OB mice (26, 102, 104, 105). Malnutrition has been empirically shown to increase the severity of IAV disease and impair NK cell function in WT mice, yet DIO and OB mice show increased NK cell infiltration of the lung at days 6 and 14 p.i., whereas DB mice show a decrease in NK cells in the BALF at day 4 p.i. (27, 69, 106). No studies have investigated the impact of obesity on the response of basophils, eosinophils, and other leukocytes during IAV infection. However, as obesity causes perturbation in the circulating leptin and adiponectin levels, leading to a state of leptin-resistance, and leptin can interact with signal transduction pathways critical to the aforementioned leukocytes and other immune cells, the impact of the obesogenic environment may extend beyond our current knowledge (87).

### Adaptive Immune Responses

Once primed by the earlier innate response, lymphocytes of the adaptive arm of the immune system can continue the control and clearance of IAV, leading to the resolution of the infection, however, obesity dampens and delays these processes (28, 107). Both T cell and B cell responses show reduced efficacy in the obese host, impairing the resolution of IAV infection.

#### T Cells

T cell lymphopenia as well as reduced proliferation, activation, and function of T cells has been described in DB and OB mice (78, 100, 108). One study with a small sample size found no changes in DIO mouse T cell proliferation and cytokine production upon stimulation *ex vivo* (98). Recent studies have aimed to clarify the literature and to elucidate the molecular mechanism behind the reduced T cell response seen in both obese mice and humans, as well as how specific T cell subsets respond to the obese environment and IAV infection.

Deficiencies in the activation and function of CD4+ and CD8+ T cells were found in studies performed with PBMCs isolated from healthy weight, overweight, and obese humans (29, 109). After *ex vivo* stimulation with H1N1 virus, CD8+, and CD4+ T cells derived from obese subjects produced significantly more IL-5, whereas IFN-γ production trended lower, compared to T cells derived from healthy weight subjects. Compared to participants of healthy weight, obese participants had a smaller percentage increase in the production of IFN-γ from activated T cells, whereas overweight and obese participants had a smaller increase in granzyme B-producing T cells (29, 110). Significantly lower fold increases in TNFα and trends toward lower fold increases in IL-6 were also described in these participants (29).

Interrogations of specific T cell subsets found no difference in the levels of αβ T cells due to obesity; however, γδ T cells were reduced in number, and those remaining showed increased differentiation that impaired their antiviral functionality and response to antigen presentation (99). Decreasing levels of γδ T cells correlates with increasing obesity status, and the remaining γδ T cells possess an immature phenotype characterized by poor IFN-γ production concordant with γδ T cells from an aged host (29, 99). The antiviral function of γδ T cells is regulated by type I IFNs, and the lack of an IFN response in the OB host could explain their poor functionality (26, 111). The poor T cell responses to influenza could also be due to reduced T cell diversity, as in DIO mice obesity accelerated the reduction in T cell receptor excision circles, leading to the reduction of T cell Vβ repertoire diversity which parallels what is seen in the aged host (112).These findings are commensurate with the “adipaging” hypothesis, whereby obese status acerbates the aging of the immune system to mirror the immune deficits seen in elderly people (53).

Obesity-related metabolic dysregulation has been proposed as the driver of poor effector T cell and helper T cell function as well as impaired memory T cell responses and vaccine efficacy, as it may alter T cell metabolism (45, 95). In their study, Rebeles et al. initially inoculated DIO or LN mice with X-31 influenza virus, then challenged DIO, LN, or weight loss diet-switched DIO mice with PR8 influenza virus (95). T cells isolated from DIO and LN mice were tested for glycolysis as measured by their extracellular acidification rate (ECAR) as well as mitochondrial respiration as measured via their oxygen consumption rate (OCR). A high OCR:ECAR ratio suggests a greater relative magnitude of oxidative phosphorylation vs. glycolysis, which is an indicator of a naïve or memory T cell phenotype as opposed to the glycolytic phenotype of effector T cells (95, 113–115). In DIO mice, as well as in DIO mice after weight loss, the OCR:ECAR ratios were elevated for both CD4+ and CD8+ T cells on day 7 of a secondary infection, showing that obesity alters the metabolic programming of memory T cells and that this change is maintained even after weight loss (95). It has been suggested that the differential cytokine milieu and growth factor deficiency blunt the maturation of antigen presenting cells, thereby extending the poor innate response seen in obese hosts to later adaptive responses. Exogenous application of IL-2 was also shown to improve the γδ T cell response, and a lack of this cytokine was suggested to impair dendritic cell antigen presentation (71, 99).

#### B Cells

Few studies have investigated how the obesogenic state affects humoral responses to IAV infection. The increased visceral adiposity due to obesity attracts increased B cell numbers, thus contributing to systemic, chronic inflammation through interactions with T cells (116, 117). Stimulated human B cells *ex vivo* secreted less IL-6, but increased IgM levels correlated with obesity state (94). However, the literature on how these baseline alterations affect the IAV-specific response is sparse.

During IAV infection, DIO mice have lower levels of H1N1-specific antibodies and a reduced neutralizing response when compared to LN mice, which is mirrored upon vaccination and subsequent homologous or heterologous challenge (44, 50). In DIO mice, un-stimulated B cells showed elevated IgG and IgM levels *ex vivo* (94). Hemagglutination inhibition (HAI) titers measured at days 7 and 21 p.i. with H1N1 virus in DIO mice were less than those in LN mice. This reduction in titer was related to a decrease in B cells in the bone marrow of DIO mice as well as to diminished specific B cell subsets (94). A single report has suggested that suggests antibody-dependent-cellular cytotoxicity (ADCC) is slightly increased in OB mice; however, no further studies have defined ADCC in the context of IAV infection (104).

To date, no studies have determined how obesity affects the response of other facets of humoral immunity, including germinal center formation, class-switching of antibodies, and the Fc receptor repertoire, to IAV infection. In other models of infection in the obese host, reduced IgG class switching but elevated IgM levels followed bacterial infection (118). DIO mice showed reduced formation of lymph node associated germinal centers and reduced recruitment of T cells and B cells to these germinal centers at day 14 p.i. (118). As leptin is a major regulator of B cell development, maturation, and activity, alterations to the adipokine balance due to obesity may directly affect B cell function, B cell interactions with other lymphoid cell types such as T-follicular helper cells, and antibody class-switching (72, 87).

## Influenza Viral Evolution in the Obese Host

Recent studies have aimed to identify not only the mechanisms by which IAV affects the obese host, but also how the obesogenic state may directly affect IAV itself. RNA viruses, with their short generation times and error-prone replication, exhibit a quasispecies population comprising viral variants that collectively increase viral fitness in the host (119). Nutritional status has been implicated in altering within-host viral evolution and has been shown to prolong infections, delay clearance, and increased shedding, all of which potentially increase viral transmission (120, 121).

### Clearance, Shedding, and Transmission

Obese patients hospitalized with severe IAV disease have higher peak viral loads and a delayed clearance when compared to non-obese patients (122, 123). Two studies have extended the findings in obese mouse models to humans by identifying high BMI as increasing the duration and quantity of IAV shedding. Maier et al. found that viral shedding is prolonged in obese adults. In their cohort of adults in Managua, Nicaragua, IAV shedding in symptomatic obese adults lasted 42% longer than in symptomatic lean counterparts. In obese adults with no symptoms or only a single symptom of active IAV infection, virus shedding lasted 104% longer than in lean adults with similar symptoms (124). No association between BMI and duration of viral shed was found for children or adolescents, or with influenza B virus (124). In a study of college-aged adults in Maryland, USA, Yan et al. quantified viral RNA in aerosols from collected breath and found that it correlated positively with BMI, which also suggests that there is increased viral load and viral shedding in obese humans (125).

Several studies have shown that WT animals clear IAV infection earlier than do OB (48, 54). In the DB model, clearance of IAV was impaired in global leptin receptor knockout mice but not in lung epithelium conditional knockout animals. This suggests that a global loss of the leptin receptor impairs viral clearance, possibly through immune cell-mediated interactions with the respiratory epithelium (69). Both epithelial dysregulation and perturbations of immune cells are observed in human and mouse obesity and may impair the elimination of IAV from the respiratory tract.

### Evolution and Population Dynamics

**Figure 2:**
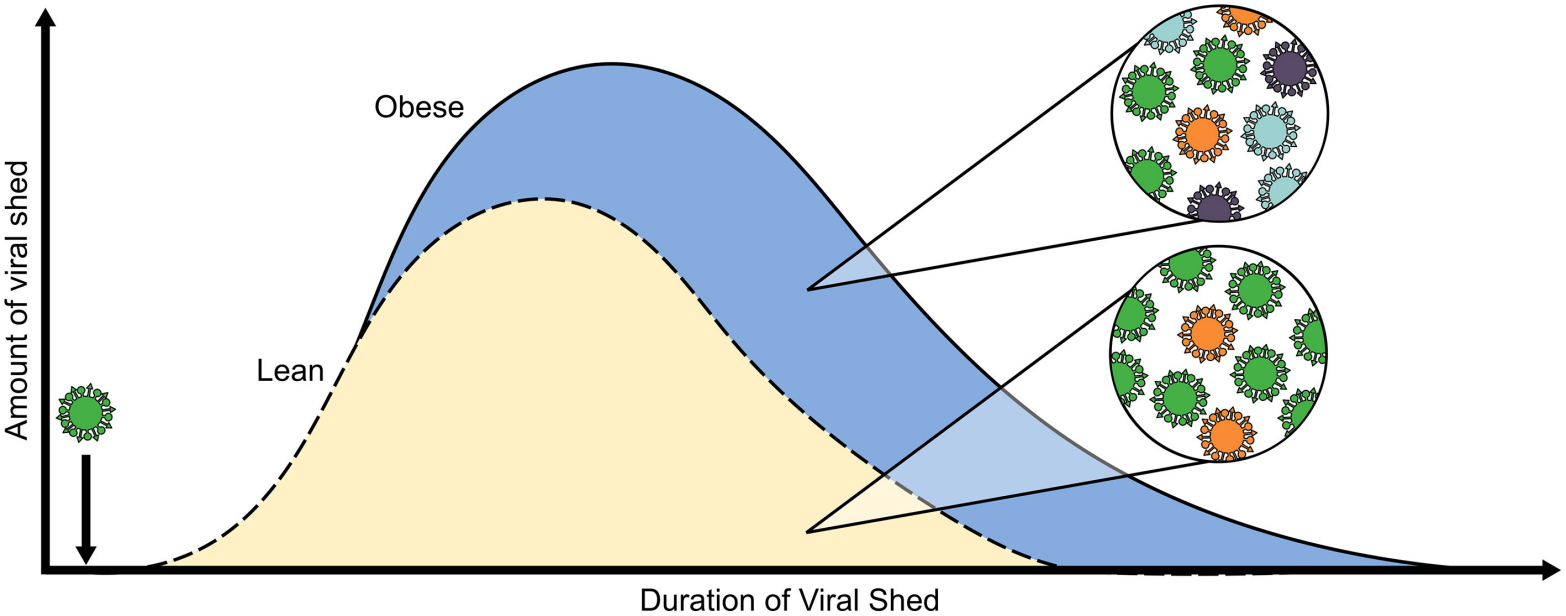
Obesity alters within-host viral population dynamics. Both the amount of viral RNA shed and well as the duration of positive samples for H1N1 virus, as determined by RT-PCR, are increased in obese adults (blue, solid line) as compared to average-weight counterparts (gold, dashed line). Furthermore, our lab determined through experimental evolution of H1N1 virus in OB and DIO mice that serial passaging through an obese host results in the emergence of minor variants that influence pathogenicity and in increased overall viral population diversity. The model is compiled from results in references (124, 125).

Host nutritional status has long been known to impact viral pathogenesis and more recently, to impact viral populations (126). However, evolutionary dynamics of IAV infection within a single host, whether human, animal models, or reservoirs, is an emerging field (127). The extended infections and increased shed seen in obese human cohorts provides ample reason to investigate how the obese host may alter IAV evolution and population dynamics (Figure 2) (124, 125).

In a study with both OB and DIO mice, we discovered the obesity promotes the emergence of virulent variants within the IAV population. We showed that inoculating naïve WT mice with viruses that had been serially passaged through OB mice resulted in enhanced morbidity compared to that in mice inoculated with virus passaged through LN or WT mice or with the parental virus. The increased virulence of the serially passaged viruses was related to point mutations in the polymerase segments and NS1, as well as to increased population-wide diversity for OB-derived viruses. This increased virulence in WT animals was observed within two rounds of passage in the obese mice, suggesting that the population changes occur rapidly. Similar passaging experiments with seasonal H3N2 virus yielded equivalent results, as the parental virus was unable to productively infect WT mice, yet the OB-passaged virus could infect and cause severe morbidity in WT mice. Furthermore, IAV propagated in normal human bronchial epithelial (NHBE) cells derived from OB donors replicated to higher titers upon inoculation of MDCK cells and caused more cell death when compared to viruses grown in NHBE cells from average-weight donors. These findings raise concerns that even a single infection of an OB host may result in an altered viral population, leading to heightened virulence properties or transmission that can affect the global evolution of IAV (127).

RNA viruses such as IAV rely on an error-prone RNA-dependent RNA polymerase; this provides a fitness advantage to the viral quasispecies by enabling the emergence of minor viral variants (119, 128). The analysis of infections with multiple variants, the rate of reassortment, and the within-host evolutionary dynamics are areas in which the impact of obesity must be defined. The increased viral spread but similar viral load observed in obese hosts as compared to lean hosts may highlight how infection of different cell types or the individual viral load within single cells can impact the cooperative and competitive dynamics within the quasispecies (127, 129). The advent of single-cell technologies and improvements in deep sequencing will usher in a new understanding of the complex interactions between host cell and IAV in lean and obese individuals (129, 130). The appearance of IAV minor variants within the individual host is concordant with the eventual emergence of the same variants in global circulation, and the impact of obesity on the rise of potentially pathogenic viral variants in the human host is an urgent need for study (127). Furthermore, certain variants may contribute to antiviral resistance, vaccine escape, and/or enhanced transmission, and the increasing prevalence of obesity will undoubtedly affect these evolutionary dynamics (127, 128, 131).

## Pharmacological Control of Influenza Virus Infection

**Figure 3:**
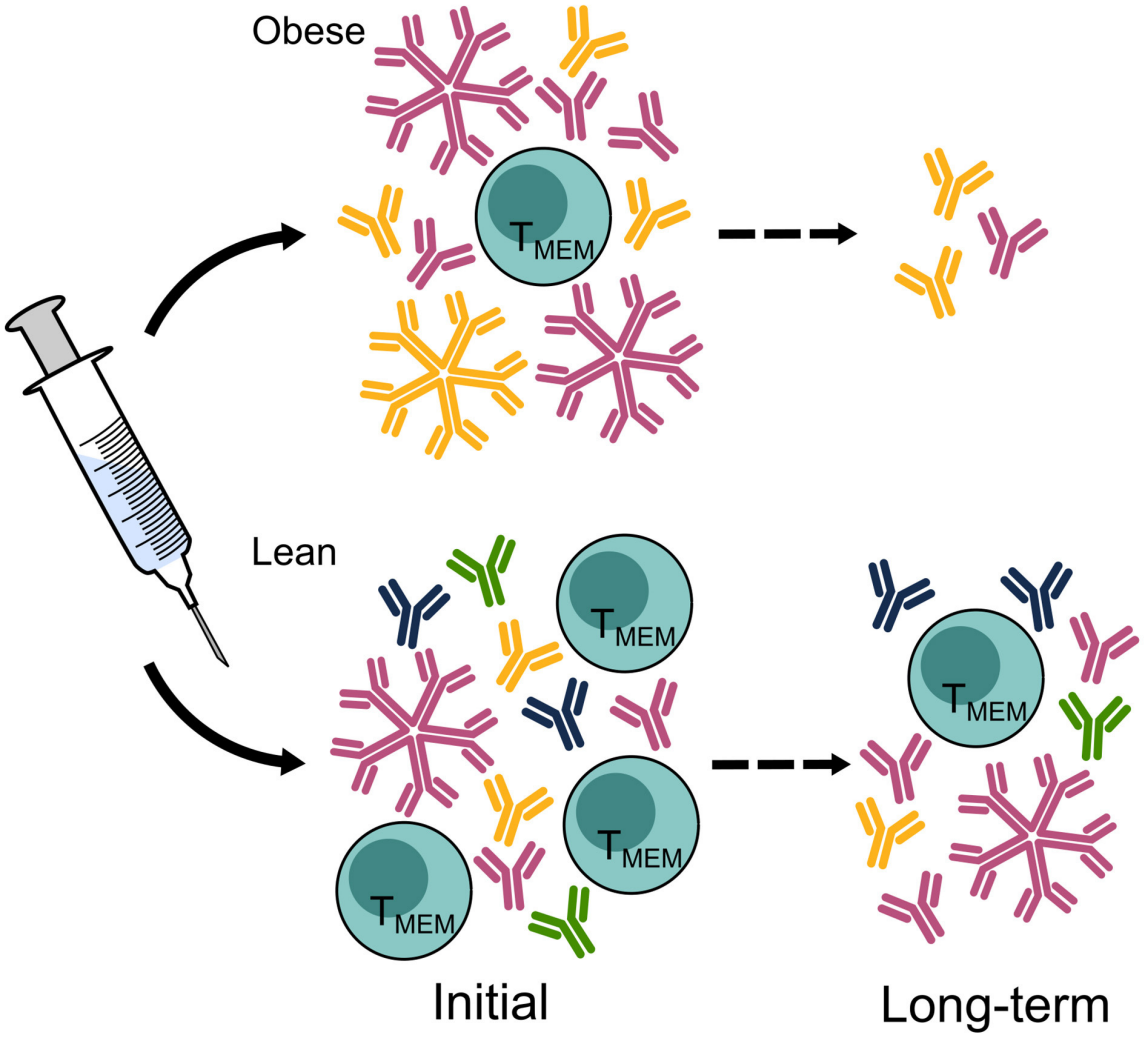
Vaccine-elicited responses are diminished in the obese host, leaving the obese population vulnerable to infection. Hemagglutination inhibition titers, the current standard correlate of protection, are equal in obese and lean hosts measured immediately after vaccination but decline more rapidly in the obese population than in the lean population. A greater breadth of HA-specific responses is elicited in lean hosts than in obese hosts, and class switching IgM to IgG may be impaired. Furthermore, the cellular immune responses to vaccination in obese hosts displays reduced activation and maintenance of memory T cells. Ab, antibody; T_MEM_, T memory cell. The figure is compiled from information in references (44, 47, 78, 110).

Vaccination before seasonal outbreaks and prompt antiviral administration during infection are key to controlling IAV infection. The poor initial and adaptive responses to natural infection and vaccination sets up an impaired ability to respond adequately to a secondary challenge. Vaccine efficacy may be decreased in obese humans, who will then rely on prompt antiviral administration to control viral infection (Figure 3). However, more studies are warranted to better understand how the obese state affects the control of infection.

### Prevention of Infection Through Vaccination

Studies of vaccine efficacy in human cohorts have shown that initial seroconversion rates are high in the obese population, but that there is a greater decline in vaccine efficacy over time than is seen in non-obese populations (132). In one study of elementary school-age children, obesity was linked to increased cough and more school days being missed. However, vaccination was equally protective for obese and non-obese children (133). Another study of adults vaccinated with the trivalent influenza vaccine found that increased BMI was associated with a reduction in the protective immune response over time (110). Although individuals with a high BMI had an initially higher fold increase in IgG antibodies and HAI titers, by 12 months post-vaccination there was a greater decline in antibody titers in obese participants than in non-obese participants (110, 134). Similar trends were seen in a childhood cohort, with the mean HAI titers against both H1N1 and type B influenza viruses being elevated overweight and obese patients (135). However, as is noted by Neidich et al. our current correlates of protection may not be predictive of actual protection from infection and may explain why vaccinated obese adults display adequate HAI titers but still succumb to IAV infection at higher rates than healthy-weight adults (132). Clearly, more studies are warranted on the population wide efficacy and waning of protection of the influenza vaccine, as well as to characterize the impact of various vaccine formulations, including the use of leptin adjuvants, in child and adult cohorts (136, 137).

Although adjuvanted vaccines perform better than non-adjuvanted vaccines in terms of the neutralizing and non-neutralizing responses elicited, both vaccines failed to provide protection against a homologous viral challenge in both OB and DIO mice (45, 47). In a heterologous challenge model with two strains of H1N1 virus, DIO mice were sub-lethally infected with H1N1 PR8 virus, then challenged with a lethal dose of H1N1 CA/09 virus. The initial PR8 virus infection provided heterologous protection against CA/09 virus in both LN and DIO mice, but DIO mice had reduced HAI and microneutralization titers as well as quicker waning of protective antibody levels and increased lung pathology (50). Analysis of the antibody-based responses revealed a reduced breadth and magnitude of HA and NA-based responses, potentiality explaining the failure to confer adequate protection (47). Even after vaccination, DIO challenge mice had higher MCP-1 and impaired B cell responses, leading to a poorer control of infection and damaging inflammatory responses (45, 94).

In addition to poor B cell responses, obese hosts exhibit poor memory T cell functions which impairs vaccine efficacy. In their study with PBMCs isolated from lean, overweight, and obese vaccinated donors, Sheridan et al. found that obese humans have reduced CD8+ T cell activation 12 months post vaccination, pointing to a reduction in vaccine efficacy in this group (110). This persists post-infection, as there is an initial reduction in influenza-specific T cells after a heterologous challenge as well as a reduction in memory T cell numbers several months post infection in DIO mice (78). However, other studies in the DIO model have found obesity to have no impact on the maintenance of pre-existing CD8+ memory T cells or the differentiation and maintenance of newly evoked memory CD8+ T cells (68).

### Control of Infection via Antiviral Drugs

Severe influenza infections can be ameliorated by the early administration of potent antiviral drugs. Continual development of antivirals to treat influenza infection is needed as historically, influenza has rapidly gained resistance to all marketed drugs (138, 139). The drugs currently approved for influenza treatment include the neuraminidase inhibitors oseltamivir, zanamivir, and peramivir as well as the recently FDA-approved endonuclease inhibitor baloxavir marboxil; the amantadines are no longer recommended because of the high resistance in circulating IAV strains (138, 140). Although these drugs have shown promise in reducing total influenza symptoms, few have been empirically tested for efficacy in obese populations.

In a prospective study of hospitalized adults, increasing awareness of the association between obesity and severe influenza virus pathogenesis may have led to quicker antiviral treatment of obese patients, thus potentially mitigating disease severity (141). However, more studies are warranted to understand how effective our current antivirals are at reaching the infected tissue and protecting obese populations from severe disease and whether the increasing reports of poor antiviral efficacy are due in part to the increasing obese population (142). A comparison of the pharmacokinetics of oseltamivir showed that obese humans clear the drug more quickly than do non-obese patients, but this increase in the clearance rate was small with potentially insignificant biological impact (143, 144). These reports suggest that dose adjustment for obese patients is not necessary from a pharmacokinetic perspective, but the question remains as to whether the dosage is appropriate to control viral replication (145, 146). In a study with DIO and OB mice, O'Brien et al. administered doses of 20 mg or 100 mg of oseltamivir carboxylate to mice 8 h before infection, with repeat doses being administered every 12 h.p.i. for 5 days (27). The 100 mg dose completely protected both DIO and OB mice, whereas the 20 mg dose increased survival from 40 to 80% in and mitigated lung pathology (27).

## Future Studies

The definition of the obese lung microenvironment and systemic alterations in the cytokine milieu continue to be refined, thereby advancing studies on how to ameliorate disease severity in the obese host. Meliopoulos et al. showed that the β6 integrin is an important regulator of the type I IFN response in the lung, in addition to controlling viral spread and post-infection wound recovery. OB and β6 integrin double knockout mice were partially protected from severe infection, showed reduced viral spread, and displayed restored levels of IFN production and signaling (54). Interventions such as weight loss programs, diet change, and exercise are the first-line of defense against the obesity epidemic, yet few studies have examined whether these interventions will improve antiviral outcomes in obese patients. Studies have found no change in cellular immunity against influenza upon weight loss in DIO mice; however, nutritional supplementation may be warranted as the administration of essential fatty acids may increase the B cell response to IAV (94, 95). Exercise improves the obese host response to IAV in the obese host, restoring levels of IAV-specific IgG antibodies, CD8+ T cells, ciliary beat frequency, IFNα gene expression and cytokine production in the BALF of DIO mice to mirror a metabolically healthy state (70). These results provide evidence that nutritional, exercise, and medical interventions may improve the collective immune response, but they highlight the continued need for development of potent antivirals and vaccines.

Continued studies of the mechanistic interactions between the lung epithelia, resident and circulating immune cells, and influenza virus will aid in the understanding of influenza pathogenesis and, potentially, in the development of novel therapeutics for obese and other high-risk populations. Modeling the collective knowledge assembled from obese mouse models, *ex vivo* human systems, and studies conducted in human cohorts may help generate new hypotheses to explain the increased disease severity and reduced immune response in obese humans. By bridging work done in virology, immunology, physiology, and metabolism, researchers can gain a holistic view of how obesity affects everything from the systemic immune response to localized events in the viral life cycle within respiratory epithelial cells.

## Conclusion

Overall, the baseline inflammatory obese state presents a barrier to the induction of a robust antiviral response. Innate and adaptive immune responses are delayed or blunted, allowing increased viral spread and extended infections in the obese host. Immunopathologic responses late in infection additionally undermine the host response, and in synergy with uncontrolled viral replication and spread, they lead to poor—often lethal—outcomes. Even when IAV infections have been cleared, the increased susceptibility to secondary bacterial infections and poor healing of the lung epithelium contribute to the high mortality rates in the obese populations. Furthermore, within the obese host influenza virus may exploit the lack of antiviral pressure, generate a more virulent population and augment disease severity, even displaying increased virulence upon subsequent infection of a lean environment.

The extent to which these findings can be extrapolated to other respiratory viruses remains unclear. The continued refinement of human primary cell cultures and the translational obese ferret model will enable a greater mechanistic understand of the impact of obesity on influenza pathogenesis, the host response, and the evolutionary dynamics of the virus (30). The growing prevalence of obesity is alarming, as infection of an obese host may soon be the standard pathogenesis, instead of the well-characterized infection of a healthy-weight host. With increasing infections in the obese host, so too may the incidence of severe influenza pandemics increase as a result of increased viral shedding, and transmission and the emergence of novel viral variants. Curbing the obesity epidemic will not only improve the quality of life for those millions of people directly affected; it will also dampen the impact of obesity on infectious disease.

## Author Contributions

RH performed the literature search, wrote and edited the drafts, created figures, and contributed to manuscript revision and submission. SS-C secured funding, provided supervision, edited the final draft, and contributed to manuscript revision and submission.

### Conflict of Interest Statement

The authors declare that the research was conducted in the absence of any commercial or financial relationships that could be construed as a potential conflict of interest.
